# Lymphadenopathy with florid lymphoid and Langerhans cell hyperplasia and hemophagocytosis mimicking lymphoma after COVID‐19 mRNA vaccination

**DOI:** 10.1002/jha2.265

**Published:** 2021-08-13

**Authors:** Suzanne Tintle, Mingyi Chen

**Affiliations:** ^1^ Department of Pathology University of Texas Southwestern Medical Center Dallas Texas USA

**Keywords:** COVID19, lymphadenopathy and lymphoid and Langerhans cell hyperplasia, vaccination

**Abbreviations**
Covid‐19: coronavirus disease 2019
EBV: Epstein‐Barr virus
HIV: human immunodeficiency virus
HLH: hemophagocytic lymphohistiocytosis
LCs: Langerhans cells
SARS‐CoV‐2: severe acute respiratory syndrome coronavirus 2

## 1 INTRODUCTION

Vaccine campaigns are currently the most promising method to combat the COVID‐19 pandemic. Messenger RNA (mRNA) coronavirus disease 2019 (Covid‐19) vaccines have been widely administered and show good immunogenicity, good tolerance, and high efficacy in inducing immune responses against SARS‐CoV‐2 [1]. Reports are rising of patients with unilateral axillary lymphadenopathy after COVID‐19 vaccination [2]. The differential diagnosis includes vaccination‐induced (post‐vaccinal) lymphadenitis and acute viral infection (particularly Epstein‐Barr virus [EBV]‐induced infectious mononucleosis, as well as rubella, human immunodeficiency virus [HIV], influenza, and herpes zoster), which can mimic malignant lymphomas [3, 4]. A thorough work‐up based on the combination of characteristic clinical, histopathological, and serologic findings is required to make the diagnosis of post‐vaccinal lymphadenitis. Establishing an accurate diagnosis can be challenging due to unusual clinical presentations or prolonged lymphadenopathy [2].

Histologically, vaccination‐induced lymphadenitis will show expansion of the paracortex with abundant immunoblasts and plasma cells and increased vascularity [3, 4, 5]. The same findings may be seen in acute viral infections or angioimmunoblastic T‐cell lymphoma [6]. The extent of these changes varies considerably from case to case, with some cases having only mild paracortical expansion and others having exuberant and atypical cellular proliferation, markedly altering the normal nodal architecture. Differences in the latency from the time of vaccination and the duration of disease at the time of biopsy may account for some of this variability. Here, we present a unique case report of florid follicular and interfollicular lymphoid and Langerhans cell (LC) hyperplasia associated with the COVID‐19 mRNA vaccine.

## 2 CASE PRESENTATION

A 23‐year‐old woman with a past medical history of asthma, eczema, and hypothyroidism developed malaise, vomiting, a fever of 104°F (40°C), axillary lymphadenopathy and acute kidney injury 1 week after receiving the second dose of the Moderna inactivated SARS‐CoV‐2 vaccine. She denied joint pain, abdominal pain, headache, or dysuria. She worked as a nurse, had no recent travel history, no pets, and no other recent incident exposures.

CT scans showed left axillary lymphadenopathy, up to 2.1 cm, and multiple enlarged lymph nodes in the abdomen. An extensive infectious work‐up was unrevealing (blood, urine and stool cultures, C. dificile, EBV serology, monospot, HIV, bartonella, tuberculosis, typus [rickettsial typhi] testing). Cytokine panel showed elevations in TNF alpha, interferon gamma, interleukin‐5, interleukin‐10, interleukin‐13, and interleukin‐6. The initial clinical impression was hemophagocytic lymphohistiocytosis (HLH)/macrophage activating syndrome versus adult Still's disease.

**FIGURE 1:**
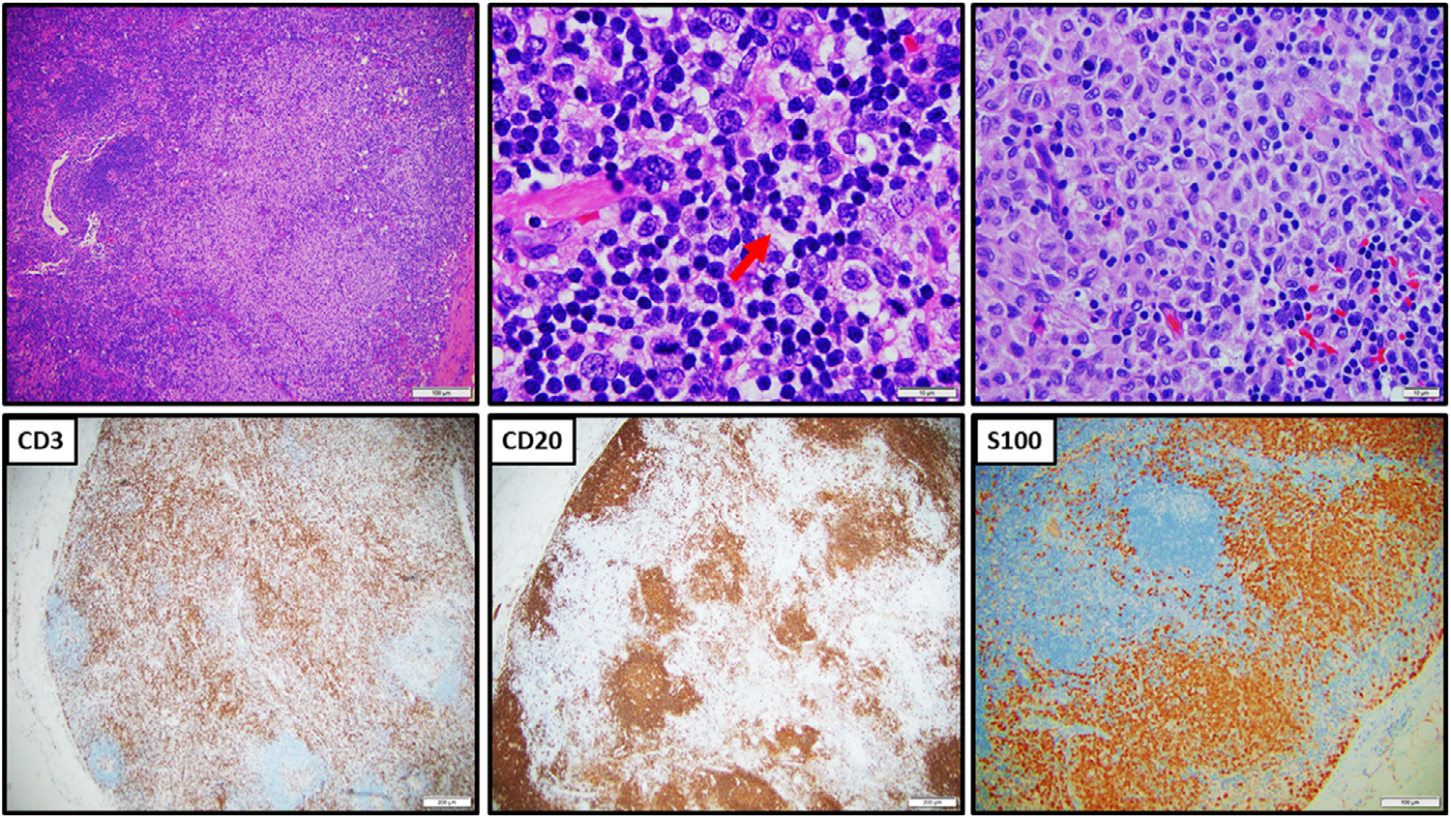
Biopsy of left axillary lymph node shows marked follicular and interfollicular hyperplasia with sinus histiocytosis and focal dendritic cells and Langerhans cells proliferation (S100+). By immunocytochemistry, there are normal T‐cells (CD3+) and B‐cells (CD20+) compartmentalization. Occasional hemophagocytosis is noted (arrow)

An excisional biopsy of the left axillary lymph node was performed. The H&E sections of the enlarged lymph nodes show hyperplastic follicles and marked paracortical expansion with polymorphic lymphohistiocytic infiltrate and hypervascularity and sinus histiocytosis. Higher magnification reveals the expansion to be caused by numerous small‐ to medium‐sized lymphocytes intermingled with many histiocytes and rare larger atypical cells. There are focal aggregates of LCs, dendritic cells, and histiocytes with rare hemophagocytosis (Figure 1).

By immunohistochemistry, the lymphoid cells are mixed T‐cells and B‐cells with normal compartment. Marked perifollicular/interfollicular T cells hyperplasia are noted with intact T‐cell antigens (positive for CD2, CD3, CD5, CD7, and mixed CD4 and CD8). There are scattered CD30+/CD20+/CD45+ immunoblasts. No Reed‐Sternberg cells or variants are identified. There is no aberrant expression of bcl‐2 in the B‐cells or expansion PD1+ T‐cells. Focal paracortical LCs proliferation is highlighted by S100 and CD1a stains. EBV in situ hybridization is negative.

Flow cytometry revealed no overt immunophenotypical aberrancy. The CD4:CD8 ratio was slightly increased (4.0:1). There was a small population (0.60%) of large CD30+ hematolymphoid cells, which included both T‐ and B‐ lineage cells, and a definite aberrant population was not identified. Notably, normal reactive immunoblasts show expression of CD30. T‐cell clonality by T‐cell receptor gene rearrangement was negative, and cytogenetics revealed a normal female karyotype (46,XX [20]). Overall, the findings are consistent with a florid reactive lymphoid proliferation.

A bone marrow biopsy was also performed and showed a normocellular marrow with active trilineage hematopoiesis. No atypical lymphoid infiltrate or hemophagocytosis was detected. No immunophenotypical abnormality was detected. After ruling out viral infections and hematolymphoid malignancy, a diagnosis of post‐vaccination reactive lymphadenitis, with paracortical/interfollicular lymphoid, LC hyperplasia, and secondary hemophagocytosis was rendered. The patient recovered rapidly with dexamethasone and anakinra within 2 weeks.

## 3 DISCUSSION

The development of mRNA vaccines for severe acute respiratory syndrome coronavirus 2 (SARS‐CoV‐2) has been confirmed to be the most effective vaccines to drive potent adaptive immune responses against COVID19 virus infection. Of the multiple epitopes on SARS‐CoV‐2, the spike (S) glycoprotein is the target commonly selected for COVID‐19 vaccine development, since it is the major SARS‐CoV‐2 surface protein and mediates viral entry by binding to the angiotensin‐converting enzyme 2 (ACE2) receptor in host cells. Enlarged lymph nodes after COVID‐19 vaccine may be mistaken for malignancy.

The administration COVID‐19 vaccine will generate ectopic S protein which is essential both for efficient loading of the vaccine into the muscle cells and for potent activation of the LCs to migrate into the lymph nodes. In the early phase of acute viral infection or vaccination, the stimulation with neoantigens will induce reactive follicular hyperplasia. Germinal center‐derived B cell responses are fundamental for the generation of high‐quality B cell responses and neutralizing antibody production [6]. As the process progresses, the follicles may become separated or disrupted by the paracortical expansion, often pushing the residual follicles to the cortex of the node. The paracortical expansion is composed predominantly T‐cells mixed with variable immunoblasts, plasma cells, plasmacytoid cells, and occasionally exuberant histiocytes, dendritic cells or LCs [4, 5, 6]. In response to cytokines stimulation in the process of subsequently encountered antigens, an increase in immuno‐stimulatory capabilities, the antigen‐activated LCs will proliferate and migrate [4]. The ultimate destination of actively migrating LCs is the paracortical regions of lymphatic nodes where presentation of peripherally acquired antigen to naïve T‐cells occurs.

Vaccine‐induced lymphadenopathy can present as a diagnostic dilemma. Sometimes the atypical cytological features, including prominent nucleoli and mitotic figures, in the proliferating immunoblasts are alarming which may raise the concern of malignancy [3]. In the later phase, apoptotic cells, focal areas of necrosis or aggregates of histiocytes manifested as scavenging hemophagocytosis can be appreciated [5].

The main differential diagnosis is between reactive paracortical/interfollicular lymphoid and LCs hyperplasia (viral/bacterial infection and/or recent immunization) versus T‐cell lymphoma [6]. It is very challenging to distinguish these entities. Clinical radiological correlation with laboratory work up is needed [5, 6, 7]. HLH can be primarily due to congenital deficiency of perforin or secondary to infection, inflammation or malignancy of lymphoma [7, 8].

There is accumulating evidence to assure the safety of mRNA coronavirus disease 2019 (Covid‐19) vaccines [1, 2]. Rare vaccination side effects may happen but usually are temporary and self‐resolving [9, 10]. The recognition of the event of lymphadenopathy associated with these vaccines is crucial to prevent misdiagnosis of lymphoma [2]. This report further emphasizes the significance of inquiry of clinical history of potentially active EBV infection or other common viruses before COVID‐19 vaccination. To mitigate the diagnostic dilemma of vaccine‐induced lymphadenopathy, patients with underlying conditions should be carefully followed up for any suspicious symptoms and signs following vaccination [1]. Documentation of vaccination status is critical to decrease unnecessary biopsies and alleviate patient anxiety.

## CONFLICT OF INTEREST

The authors declare that there is no conflict of interest.
